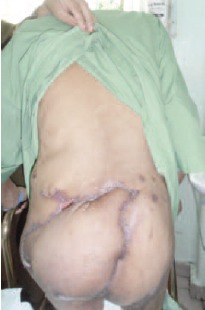# Giant Sacral Chondrosarcoma in an Elderly Male : A Case Report

**DOI:** 10.5704/MOJ.1403.007

**Published:** 2014-03

**Authors:** HZ Chan, CS Wang, A Azuhairy, A Hau, O Zulkiflee

**Affiliations:** Department of Orthopaedics, Pulau Pinang Hospital, Georgetown, Malaysia; Department of Orthopaedics, Pulau Pinang Hospital, Georgetown, Malaysia; Department of Orthopaedics, Pulau Pinang Hospital, Georgetown, Malaysia; Department of Orthopaedics, Raja Perempuan Zainab II Hospital, Kota Bharu,Malaysia; Department of Orthopaedics, Pulau Pinang Hospital, Georgetown, Malaysia

## Abstract

Primary sacral tumours are rare, therefore experience of managing their associated complications are very limited. Effective surgical treatment of pelvic chondrosarcoma remains a major challenge for orthopaedic surgeons, due to the complex anatomic structure of the pelvis, the lack of defined compartment borders, the close vicinity to vital structures, and the risk of jeopardizing pelvic structural stability. We report a rare case of a giant sacral chondrosarcoma (100cm x 80cm) in an elderly male who successfully underwent tumour resection with good functional outcome and recovery. Long term follow up is essential in view of the possibility of local tumour recurrence.

**Key Words:**

Giant Chondrosarcoma, Sacrum, Surgery, Elderly Male

## Introduction

Primary tumours of the sacrum are rare. Metastases are the most common malignant tumours of the sacrum and only six percent of all malignant bone tumours arise from sacrum, including chordomas (50% of cases), multiple myeloma (10%), lymphoma (9%), osteosarcoma (4%), and in very rare circumstances chondrosarcoma involve sacrum^1^ (2%). Chondrosarcoma predominantly arises in elderly patients with a peak incidence in the sixth decade. It has a predilection for shoulder, pelvis, proximal femur, costal region and rarely involves sacrum^2^. Secondary chondrosarcoma can develop from benign lesions such as enchondromatosis or with single or multiple cartilaginous osteochondromas in Maffuci syndrome and Ollier’s disease. Clinical occurrence of chondrosarcoma varies widely. Lowgrade chondrosarcoma often grow slowly with a very small rate of distant metastases. On the other hand, high-grade, mesenchymal and dediffrentiated chondrosarcoma are highly malignant bone tumours with a poor prognosis. Tumour relapses are strictly related to histologic grade^3^. In general, surgery is the most important therapeutic modality because these tumours are generally not sensitive to chemotherapy and radiotherapy treatment.

## CASE REPORT

A 62-year old male, presented with painless gluteal swelling for the past 8 years. The swelling was gradually enlarging over the years. Due to the size and location of tumour, the patient was only able to stand with support and sleep in a sitting position for the past one year (Figure 1a) and (Figure 1b). Two months prior to consultation the patient started to have severe gluteal pain which radiated down both legs and ulceration with haemoserous discharge. The patient appeared cachexic and clinical examinations revealed a 100cm x 80cm multilobulated mass arising from the gluteal and extending to lumbosacral region. The mass was hard in consistency with dilated veins on its surface. He had right foot drop. No lymph nodes were palpable and peripheral pulses of lower limbs were present. Computed tomography scan reported a large lobulated enhancing mass arising from the sacrum with gluteal muscle invasion, bony destruction and erosion into sacral spinal canal (Figure 2a). It extended superiorly into the left erector spinae muscle and displaced rectum anteriorly with focal areas of loss of fat plane. However there were no distant metastases. Trucut biopsy of the mass showed abundant hypocellular avascular hyaline chondroid matrix separated by fibrocollagenous tissue with malignant chondrocytes embedded within lacuna, which is consistent with well-differentiated chondrosacroma (Figure 2b).

A multidisciplinary intervention by orthopaedic surgeon, plastic surgeon, radiologist, and anaesthetist as well as oncology team was commenced and the patient successfully underwent tumour excision. Intra-operative by the tumour mass was noted to have originated from S1 down to the coccyx and mid buttock and encased the left sciatic nerve on the left side. Anteriorly, there was presence of a multilobulated mass extending into the pelvis with necrotic material not involving the anterior bladder wall or the rectum. A total of 27 kg of tumour mass was debulked leaving a base of tumour of 40 x 30cm which required the plastic surgery team to excise with wound reconstruction and coverage. (Figure 3a) Defunctioning colostomy was created to attain good perianal hygiene due to the close proximity of the surgical wound. Post-operatively, wound breakdown occurred. The patient was kept in the ward for dressing and intravenous antibiotics until the wound fully healed. In view of the massive tumour size, the patient was unable to undergo Magnetic Resonance Imaging (MRI) of the pelvis for tumour staging preoperatively. MRI of the pelvis done postoperatively reported residual tumour overlying the sacrum with extension into the pelvis and gluteal region. The patient was referred to oncology for radiotherapy in view of residual intra pelvic tumour. On follow up review of the patient at six months, one year and one and a half years, the patient showed no local recurrence of the tumour and the scar was well-healed (Figure 3b). He was able to ambulate well and had no bowel or urinary dysfunction. He eventually regained a good quality of life as he had since been able to sleep flat and was pain free.

## Discussion

The late presentation of this elderly man to doctors is because of the insidious onset of pelvic chondrosarcoma with relatively symptom- free presentation at early stage and absent distant metastasis. Therefore, sacral tumours are often too large for achieving adequate surgical margins during resection as it remains clinically silent for a long time before the patients finally decide to pay a visit to the doctors when they start to experience local pain due to its mass effect and compression. In this case, the huge size of the sacral mass with ulceration and haemoserous discharge, neurological deficit of right leg, CT scan report of massive bony destruction and intra pelvic involvement of tumour all directing towards the possibility of a highly malignant tumour which is technically demanding to resect, especially without the aid of MRI to delineate the extension of tumour and neurovascular involvement. Due to the enormous size of the sacral tumour, we encountered difficulty to fit the patient into the MRI machine. Wide excision of sacral chondrosarcoma with adequate surgical margin remains the procedure of choice in chondrosarcoma treatment, since this is the most effective way of reducing tumour recurrence rate4. As a result, it is important to get early diagnosis and proceed with wide excision aiming to achieve as clear margin as possible because surgical approaches are often limited by the size of the tumour and the proximity to vital structures in advanced cases. The surgeon has to maintain a subtle balance between ensuring adequate resection margins and the risk of endangering adjacent vital structures as well as the structural stability of the pelvis. In our case as adequate surgical margins were difficult to achieve, only tumour debulking surgery was possible because of the extensive involvement of sacral plexus and intra pelvic structures. Although chondrosarcomas are reported to have relatively low sensitivity to chemotherapy and radiotherapy, local control is sometimes achieved through chemoradiation. In this patient debulking surgery in a massive sacral chondrosarcoma combined with radiotherapy was an option for local control of low grade well-differentiated chondrosarcoma to improve patient quality of life and survival rate.

**Fig. 1a: Giant sacral chondrosarcoma:**
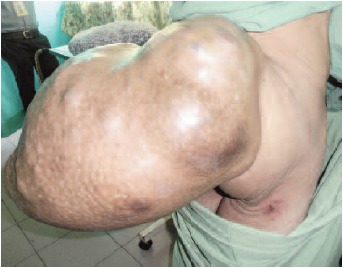


**Fig. 1b: Patient unable to sleep in supine position due to the sacral mass.**
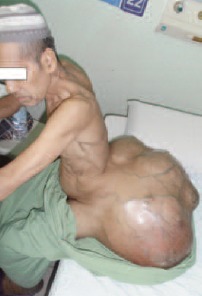


**Fig. 2a: CT pelvis, sagittal view shows huge sacral mass with bony destruction and intrapelvic involvement.**
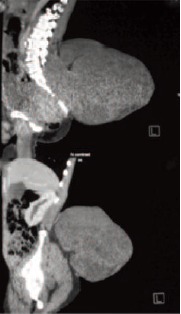


**Fig. 2b: Excised tumour:**
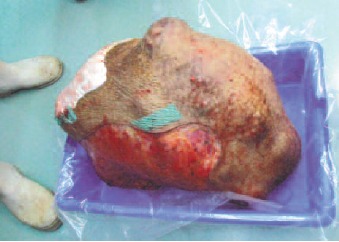


**Fig. 3a: High power magnification of tumour showing clusters of malignant chondrocytes within lacunae (mitotic figures and prominent nucleolus).**
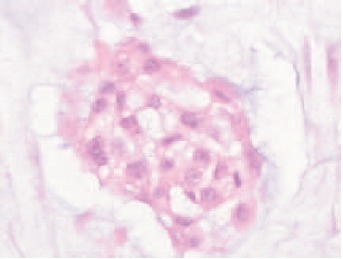


**Fig. 3b: 6 weeks post tumour excision.**